# Writing Together to Get AHEAD: an interprofessional boot camp to support scholarly writing in the health professions

**DOI:** 10.5195/jmla.2017.222

**Published:** 2017-04

**Authors:** Megan von Isenburg, Linda S. Lee, Marilyn H. Oermann

## Abstract

**Background:**

Writing for publication is an integral skill for both sharing research findings and career advancement, yet many faculty lack expertise, support, and time to author scholarly publications. Health professions educators identified writing as an area in which a new educators’ academy could offer support.

**Case Presentation:**

To address this need, a writing task force was formed consisting of a librarian, a School of Medicine faculty member, and a School of Nursing faculty member. The task force launched two initiatives to motivate and support faculty writing and publication over two academic years. In the first year, a structured interprofessional “boot camp” consisting of a sequenced, modularized approach to manuscript completion was offered. In the second year, community building, in-person writing sessions, and incentives were added to the structured tasks. In year one, twenty participants enlisted in the boot camp, nine of whom completed a manuscript for submission by the end of the program. Qualitative feedback indicated potential improvements, which were put in place in the second program. In year two, twenty-eight participants enrolled, and eleven submitted thirteen manuscripts for publication by the end of the program.

**Conclusions:**

Structured tasks, frequent deadlines, and professional editorial assistance were highly valued by participants. Time remains a barrier for faculty seeking to complete manuscripts. As experts in many facets of the publication process, librarians are well positioned to partner with others to facilitate faculty and staff development in writing.

## BACKGROUND

Disseminating research findings and best practice evidence through journals and other publication venues is critical to expand knowledge and improve patient care. In addition, career advancement for faculty in health professions schools and other academic settings requires production of scientific peer-reviewed publications. However, many health care professionals have limited backgrounds in scientific writing and lack support for writing. Faculty and health sciences librarians’ efforts to foster writing skills among graduate students, health professions trainees and faculty, and clinicians encompass a variety of strategies. Some of these strategies are intended to develop or improve the individual’s writing skills, such as including writing assignments for students in their academic coursework, writing across the curriculum, holding workshops to help graduate students improve their writing skills, and critiquing scholarly writing by faculty and peers with feedback [1–3].

Other strategies focus on writing for publication and preparing faculty and clinicians to develop manuscripts and navigate the publishing process. These include retreats that provide concentrated time and guidance to complete a manuscript, workshops that describe the process of writing for publication, faculty development programs that provide some protected time for clinician educators to prepare their manuscripts, and writing groups [4–10]. Writing groups not only guide faculty and clinicians in preparing a manuscript, but also provide the support that they need to complete it. These groups can be formal and highly structured or more informal. For example, Brandon et al. described their “Writers’ Circle” to promote scholarship and support radiology faculty members in revising previously rejected manuscripts. After an initial meeting, interactions tended to be informal, both in person during clinical work and online [11]. Also, Steinert et al. described their use of a half-day workshop, three peer writing groups, and independent study guided by a self-paced workbook to assist faculty in writing about their educational innovations in medicine [12].

When the goal is to encourage writing for publication, strategies are more effective if they are combined with individualized coaching and mentoring for the faculty member or clinician [2, 3, 13, 14]. The coaching and mentoring can be provided in person or via telephone and email [11]. This support is critical to lead faculty and clinicians through each step of the process of preparing their manuscripts and to encourage them to complete their papers, considering competing demands on their time.

Librarians are well positioned to offer writing support, since they typically already assist faculty, students, and other staff with many aspects of the publication process, including identifying relevant sources for a literature review, identifying appropriate journals for manuscript submission, and complying with public access policies from the National Institutes of Health (NIH) and other funding agencies. Librarians also teach bibliographic style to students [15, 16] and assist them with developing skills in using bibliographic management software.

## STUDY PURPOSE

Although many articles describe strategies to prepare physicians, nurses, and other health professionals to write for publication, the interventions are commonly designed for clinicians and faculty in specific professions and are not interprofessional. This paper describes and reports the outcomes of two initiatives undertaken by Duke Health’s educators’ academy, the Academy of Health Education and Academic Development (AHEAD), to promote dissemination of educational scholarship by health professions faculty and clinicians. Our writing intervention, which included two structured programs designed to educate and motivate participants, was interprofessional and included faculty and clinicians from medicine, nursing, physical therapy, and public health.

## CASE PRESENTATION

### 2014–2015 Writing Boot Camp

In 2014, Duke AHEAD tasked a small group consisting of a librarian and faculty members from the Schools of Medicine and Nursing to develop a mechanism to increase writing support for health professions faculty, trainees, and clinicians. The task force developed and launched an initiative to motivate and support their writing for publication. This began with a one-hour panel discussion on writing topics that was open to the entire Duke Health community. Attendees represented multiple health professions, including medicine, nursing, physical therapy, and public health. Approximately twenty attended the panel discussion. The panel was led by the associate dean for library sciences and archives at Duke University School of Medicine and the executive editor of the *American Heart Journal.* Presenters discussed developing good writing habits, overcoming writer’s block, selecting the right journal, and understanding open access.

Following this panel discussion, the task force launched a structured “Writing Boot Camp,” designed to motivate participants through a sequenced approach to manuscript completion that included specific writing tasks with deadlines, small groups, and peer feedback over a four-month period. Participants were recruited through advertisements on the Duke AHEAD website and emails to members of Duke AHEAD. At the start of the boot camp, nineteen participants—including fourteen faculty, one house staff, three doctoral nursing students, and one medical student—met face-to-face in a conference room in the medical library. Following introductions and presentations on their manuscript topics, the participants were given a schedule of specific writing tasks with due dates over a twelve-week period.

Participants were grouped into five teams and assigned a guide (one of the task force members), based on their writing topics. The librarian guided one of the groups and provided support for all of the participants related to literature searches, available resources on the medical library website, and other resources that individual participants requested. The librarian also gathered tips on writing for publication, which she shared with participants via email.

Groups were asked to be accountable to one another by letting each other know when tasks were completed and by reading each other’s completed manuscripts. Guides were intended to be shepherds who were available to answer questions, provide resources, and keep people moving toward manuscript completion. At the end of this boot camp, four of the nineteen participants had withdrawn, nine participants had submitted nine manuscripts for publication, and six participants had manuscripts in progress.

Following boot camp completion, the task force invited participants to attend a focus group to provide feedback on the structure of the writing program and suggestions for future interprofessional writing groups. Four participants attended the focus group, shared their ideas about the boot camp, and offered suggestions for future writing groups. This focus group was held in person. Having structured tasks and deadlines was highly valued by all. Getting peer feedback from group members, which was not always completed, was recognized as problematic due to participants’ time constraints. Suggestions for improvement included offering monthly meetings, repeating the boot camp program throughout the year, creating more frequent deadlines for manuscripts, financing professional editorial services for participants, and providing a dedicated space and time for writing.

### 2016 Writing Together to Get AHEAD Program

Following the 2014–2015 boot camp, the Duke AHEAD writing task force members applied for and received funding from the educators’ academy to develop a new program that addressed many of the comments and concerns from participants of the initial Writing Boot Camp. The “Writing Together to Get AHEAD” intervention, which took place over four months at the beginning of 2016, provided a semi-structured program with incentives in the form of vouchers for editorial services. Because progress information was collected from participants regularly via surveys, this project was reviewed and determined to be exempt by the Duke University Health System Institutional Review Board.

Similar to the boot camp, the Writing Together program began with individual and small-group in-person orientations to the writing program led by the librarian and the School of Nursing faculty member. This program offered a breakdown of key writing tasks and deadlines (Table 1); however, in the second program, these deadlines were more flexible and could be extended over a sixteen-week period (rather than a twelve-week period). Similar to the boot camp, participants were recruited through advertisements on the Duke AHEAD website and emails to members of Duke AHEAD. Participants were required to work on education-themed, as opposed to clinical, manuscripts. This restriction was included in the Writing Together program consistent with the goal of Duke AHEAD to increase educational scholarship throughout the health system. A total of twenty-eight health professions faculty from medicine, nursing, physical therapy, and public health; two staff nurse educators; and one physical therapist registered for the program. Participants were not assigned to a group but could choose to work with a partner, as participants in the first boot camp recommended not assigning small groups but instead allowing faculty and clinicians to select their own writing partners or to work in a small group.

**Table 1:**
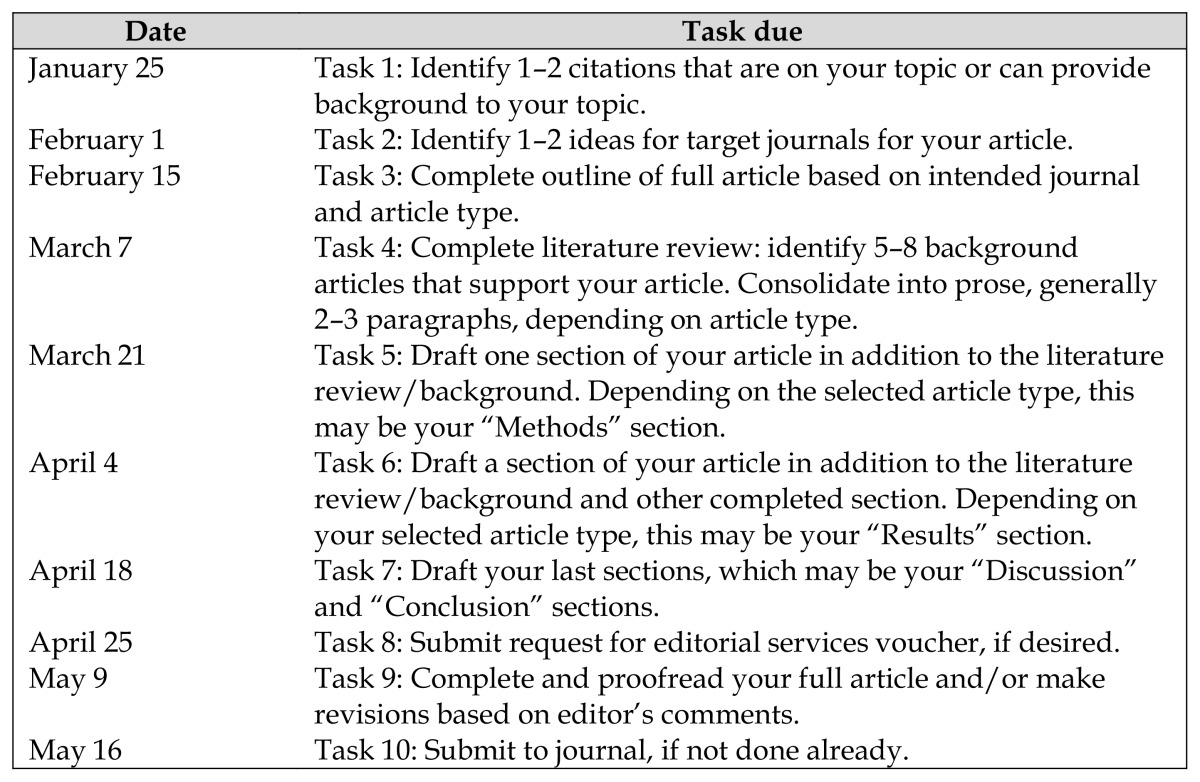
Writing tasks and sample dates

Date	Task due
January 25	Task 1: Identify 1–2 citations that are on your topic or can provide background to your topic.
February 1	Task 2: Identify 1–2 ideas for target journals for your article.
February 15	Task 3: Complete outline of full article based on intended journal and article type.
March 7	Task 4: Complete literature review: identify 5–8 background articles that support your article. Consolidate into prose, generally 2–3 paragraphs, depending on article type.
March 21	Task 5: Draft one section of your article in addition to the literature review/background. Depending on the selected article type, this may be your “Methods” section.
April 4	Task 6: Draft a section of your article in addition to the literature review/background and other completed section. Depending on your selected article type, this may be your “Results” section.
April 18	Task 7: Draft your last sections, which may be your “Discussion” and “Conclusion” sections.
April 25	Task 8: Submit request for editorial services voucher, if desired.
May 9	Task 9: Complete and proofread your full article and/or make revisions based on editor’s comments.
May 16	Task 10: Submit to journal, if not done already.

Weekly “Shut Up and Write Sessions” were offered in quiet spaces around the medical center and were designed to give participants a built-in time dedicated to writing. Each session was scheduled for a two-hour block of time. No email, pagers, phone calls, or similar interruptions were allowed. Participants each volunteered to “champion” a week. This involved emailing the participants with the writing task for the week, a reminder about the time and location for the “Shut Up and Write Session,” and a writing tip. A web page was developed by the librarian on the task force and was added to the medical center library’s website. The web page provided information about the writing program and its components and included resources on writing in the health fields. Participants were sent an email alerting them to the web page, and a link to the web page was included on the Duke AHEAD website.

Monthly progress surveys were sent to the participants to gauge their completion of tasks. Upon manuscript completion, participants could request free editorial services from an editorial group located in the university, which typically charges a fee to faculty and clinicians.

Of the 31 participants, 24 reported on their progress in the program in the final evaluation survey. Most completed the initial writing tasks: identify 1–2 citations on their topic (n=19, 79%), select potential target journals (n=20, 83%), prepare an outline (n=18, 75%), and complete the literature review (n=17, 71%). As time went on, fewer participants reported completing drafts of sections of their manuscript, with 63% (n=15) completing 1 section and 54% (n=13) completing more than 1 section. By the end of the 16-week program, 11 participants submitted 13 manuscripts to journals. They spent a mean of 3.3 hours per week on their writing tasks. Ten of the participants used a partner to keep them on target with their writing.

Participants reported the comparative value and utilization of the various program components on the final evaluation. The most valued components of the Writing Program were breaking writing into structured tasks, having deadlines, and offering vouchers for editorial services (Table 2). The “Shut Up and Write Sessions,” while recommended by participants in the first boot camp, were not highly valued or well attended. Of the 12 scheduled sessions, 6 participants each attended 1 session, and 3 attended 2 sessions. Vouchers for editorial services were the second most-valued component of the writing program and were used for editing 10 of the manuscripts. Eight participants redeemed vouchers, 1 of whom used editorial services for 3 manuscripts. The mean number of hours that editors spent editing a manuscript was 9.47, ranging from 2.9 to 14 hours per paper.

**Table 2:**
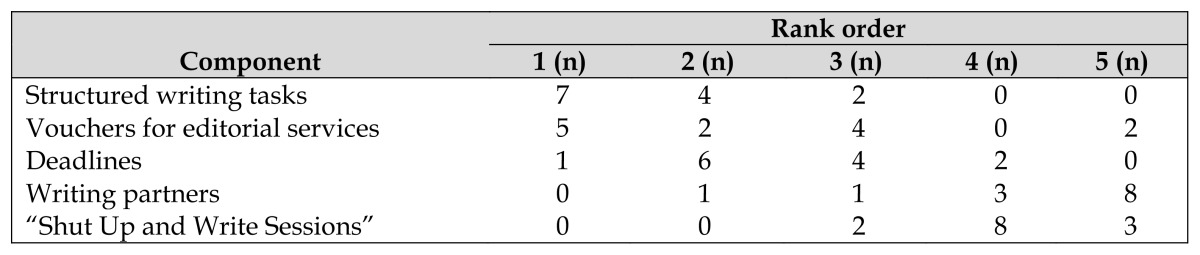
Valued components of writing program

	Rank order				
	
Component	1 (n)	2 (n)	3 (n)	4 (n)	5 (n)
Structured writing tasks	7	4	2	0	0
Vouchers for editorial services	5	2	4	0	2
Deadlines	1	6	4	2	0
Writing partners	0	1	1	3	8
“Shut Up and Write Sessions”	0	0	2	8	3

Sixteen participants identified barriers that prevented them from making progress on their writing tasks. The most common barrier was a lack of time because of workload and other work-related responsibilities (n=13, 81.3%). Other barriers, each identified by at least 1 participant, were the complexity of the publication process, competing scholarly requirements such as grant submissions, and family commitments.

## DISCUSSION

In the Writing Boot Camp, 60% of the participants submitted manuscripts by the end of the program. The second program had a larger number of participants with a lower percent of submissions (35.5%), but the percent was still higher than some previous reports. For example, Richardson and Carrick-Sen offered a writing for publication program for nurses and other health professionals, and only 12 of 50 participants in their program submitted manuscripts [17]. The second writing program addressed many participants’ concerns from the first program, specifically more flexibility in completing tasks, a dedicated time and space to write, and professional editorial services at no cost. However, some of these components remained underutilized.

Time was clearly a barrier for many participants. Among clinician educators, barriers to scholarship included a lack of time and skills in writing for publication as well as limited mentoring and support [10]. Clinicians and health professions faculty frequently lack flexibility in their schedules due to patient care and teaching obligations. It was, therefore, not surprising that it was difficult for them to attend our “Shut Up and Write Sessions.” It was surprising, however, that the opportunity to receive professional editorial services at no cost was not a strong enough incentive to complete the manuscripts within the four-month program. Time was also an issue for our Writing Boot Camp guides, as it has been for others offering writing support services [16, 17]. In the initial program, each guide shepherded a small group of writers, which involved reading and providing feedback on several papers multiple times. This consumed a significant amount of time that was off-loaded to the editorial services group in the second program.

Timing of the program in the context of other work and family responsibilities was also a problem. Several participants reported that competing work priorities and nonresponsive coauthors derailed their progress. Offering a program that was more frequent or had more flexible start dates could address this concern. The issue with competing work priorities is difficult to resolve without release time for participants [10]. Derouin et al. recommended that clinicians receive incentives such as paid and protected time to attend writing workshops and complete their manuscripts [3].

The authors’ intention in creating a timeline of smaller tasks and regular deadlines was to provide motivation and a path through what is frequently considered a daunting project. Participant feedback was very positive for these smaller, structured tasks with frequent deadlines and personal reminders. One participant commented, “the email reminders were helpful. Even when I saw Shut up and Write in a[n email] subject line, it spurred me to...shut up and write! I mostly needed motivation and encouragement.”

Overall, we believe that writing support programs can be successful in motivating and providing guidance to faculty and clinicians in the health professions. These two writing programs also demonstrate an important and evolving role for health sciences librarians in educating faculty and clinicians on writing for publication and health professions educational scholarship. The panel presentation for our Writing Boot Camp was led by a librarian, and the task force that planned and led both writing programs included a librarian who, together with the two faculty members, provided programmatic planning, participant guidance, and resources to participants. The writing programs also demonstrated successful interprofessional collaborations to promote educational scholarship among health professions faculty and clinicians. Our next steps are to analyze the processes used by our most successful participants to identify what personal techniques enabled them to complete their manuscripts. We hope to integrate these into an evolving interprofessional writing support program that is grounded in motivation, encouragement, mentoring, and editorial assistance.
